# Application of *Bacillus thuringiensis* strains with conjugal and mobilizing capability drives gene transmissibility within *Bacillus cereus* group populations in confined habitats

**DOI:** 10.1186/s12866-020-02047-4

**Published:** 2020-11-26

**Authors:** Xiaomin Hu, Doudou Huang, Joseph Ogalo, Peiling Geng, Zhiming Yuan, Hairong Xiong, Xiaofu Wan, Jiahui Sun

**Affiliations:** 1 College of Life Science, South-Central University for Nationalities, Wuhan, 430074 China; 2 Wuhan Institute of Virology, Chinese Academy of Sciences, Wuhan, 430071 China

**Keywords:** *Bacillus thuringiensis*, *Bacillus cereus* group, Plasmid, Conjugation, Mobility, Population

## Abstract

**Background:**

*Bacillus thuringiensis* bacteria share similar genetic, physiological, and biochemical characteristics with other members of the *Bacillus cereus* group. Their diversity and entomopathogenic origin are related to their mobile genetic elements. However, the effects of wide-spread application of *B. thuringiensis*-based pesticides on genetically related *B. cereus* group populations present in the environment remain poorly understood.

**Results:**

We first identified pBMB76 from *B. thuringiensis tenebrionis* as a new conjugative plasmid. Mixed mating experiments suggested that pBMB76 may compete with pHT73, another known conjugative plasmid. Applications of single (*tenebrionis* 4AA1 and *kurstaki* HD73 carrying pBMB76 and pHT73, respectively) and mixed (4AA1 + HD73) *B. thuringiensis* strains were performed in confined plot habitats (soil and leaf) over two planting seasons. In total, 684 *B. cereus* group isolates were randomly selected from different treatment sets, and the transmissibility and occurrence rate of potential conjugative plasmids were surveyed. Results showed that the percentage of isolates with plasmid mobility was markedly enhanced in the *B. thuringiensis*-sprayed groups. Furthermore, we performed multi-locus sequence typing (MLST) for a subset of 291 isolates, which indicated that the dominant sequence types in the treated habitats were identical or related to the corresponding sprayed formulations.

**Conclusions:**

The application of *B. thuringiensis* strains with conjugal and mobilizing capability drove gene transmissibility within the *B. cereus* group populations in confined habitats and potentially modified the population structure.

**Supplementary Information:**

The online version contains supplementary material available at 10.1186/s12866-020-02047-4.

## Background

Based on a newly proposed average nucleotide identity genomospecies threshold (92.5 ANI), the *Bacillus cereus* group currently consists of 12 species, including *Bacillus pseudomycoides, Bacillus paramycoides, Bacillus mosaicus, Bacillus cereus* sensu stricto*, Bacillus toyonensis, Bacillus mycoides, Bacillus cytotoxicus, Bacillus luti*, *Bacillus bingmayongensis, Bacillus gaemokensis, Bacillus manliponensis*, and *Bacillus clarus* [1]. However, the distinction of notable group members, e.g., entomopathogenic *B. thuringiensis*, opportunistic *B. cereus*, and bioterrorism agent *B. anthracis*, still follows conventional insecticidal or pathogenic criteria. Linking genomospecies and phenotype in the diagnosis of *B. cereus* group species has produced many incongruences. This is thought to be related to the genetic determinants of phenotype traits, which are mostly located on mobile genetic elements (MGEs) (e.g., plasmids, phages, transposons, and insert elements) with the capability of horizontal gene transfer (HGT) [2].

The HGT of MGEs between *B. thuringiensis* and *B. cereus* group relatives has been reported in previous studies [3–5]. In addition, it has been suggested that secreted virulence factors may act as cooperative public goods within a strong spatial structure, and HGT may promote cooperation within hosts [6]. Certainly, HGT can help expand virulence or adaptation factors within non-host habitats, thereby enriching genetic diversity and allowing transfer among intra-members of the *B. cereus* group within the natural environment [7]. This may explain why the high-throughput *in-silico* ANI approach has identified an increasing number of *B. cereus* group species but struggled to detect congruences between the genomospecies definition and phenotype in this group.

*Bacillus thuringiensis*-based pesticides represent over 50% of current microbial pesticides employed in integrated pest management [8]. Most isolates developed as pesticides, such as *kurstaki* HD1 and *israelensis* 4Q2, display conjugal and mobilizing capability [9, 10]. Furthermore, the HGT of MGEs not only occurs under laboratory conditions [11] but also in natural habitats, e.g., soil, folia, or insect midgut [9, 12, 13]. This raises the question of whether the application of *B. thuringiensis*-based pesticides with conjugal and mobilizing elements can promote gene flow within *B. cereus* group populations and enhance MGE dominance, and thus affect community composition in the treated area, especially confined habitats.

**Fig. 1:**
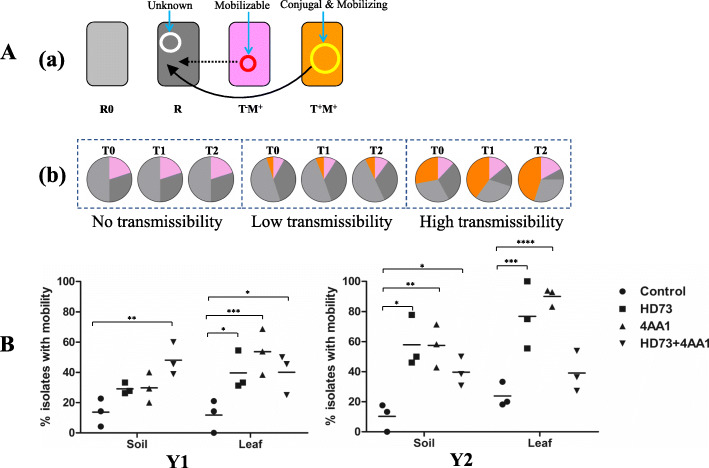
Effects of pesticide application on genetic transmissibility within *B. cereus* group populations in sprayed plots. **A:** Ideal model of self-transmissibility and mobility within *B. cereus* group in confined niches. (a): Classification of isolates based on transmissibility. Orange: isolate with self-conjugative and mobilizing capability (T^+^M^+^); pink: isolate with mobilization but no self-conjugative capability (T^−^M^+^); dark gray: potential host (R); light gray: non-host (R0); (b): Proposed changes in genetic population in a confined plot with or without transmissibility. **B:** Effects of pesticide application on genetic transmissibility within *B. cereus* group populations in 2017 (Y1) and 2018 (Y2). Non-conjugative but mobilizable plasmid pBC16 carrying tetracycline (Tet) resistance gene was used as an indicator for transmissibility and mobility. Transconjugants containing mobilizable plasmids were first screened on double-resistant plates. Primer pairs specific to chromosomal background of recipient and plasmid pBC16, respectively, were used to confirm correct transconjugants. Each mating experiment was repeated three times and isolates with > 2 positive results were defined as having mobility. In total, 684 isolates (ca. 40/set) were tested in mobility experiments. *P <* 0.1: *; *P <* 0.01: **; *P <* 0.001: ***; *P <* 0.0001: ****

The *B. cereus* group isolates can be divided into at least four types. Some isolates carry MGEs with self-transfer and mobilization capabilities, i.e., can transfer themselves and also activate “mobilizable” plasmids to move intercellularly (defined as T^+^M^+^-type strains). Generally, mobilizable plasmids carry a mobilization gene (*mob*) and *cis*-acting origin-of-transfer site (*oriT)* but lack certain genetic determinants (e.g., genes encoding sex pili in gram-negative bacteria or diverse surface components in gram-positive bacteria) required to establish a transport channel, and therefore may utilize a channel built by a conjugative plasmid to complete transfer [14]. *Bacillus thuringiensis* serovar *israelensis*, which carries the pXO16 plasmid, is considered a typical T^+^M^+^ representative. The pXO16 plasmid can “circulate” among other *B. cereus* group members within a broad host range, and not only demonstrates efficient self-transfer but also the capability to mobilize certain non-conjugative plasmids (e.g., pBC16 and pUB110) and transfer chromosomal loci [3–5]. Some isolates that carry mobilizable elements cannot self-transfer (defined as T^−^M^+^-type strains) but can be mobilized by T^+^M^+^-type strains [5, 14]. In addition, some isolates can accept heterologous conjugative or mobilizable plasmids as potential hosts (R), whereas others cannot (R0) [15, 16]. It has been hypothesized that if only T^−^M^+^, R, and R0 exist within a habitat, then genetic exchange will not occur, and the genetic structure will remain unchanged. In contrast, if T^+^M^+^-type strains also exists within a habitat, then, under appropriate conditions (e.g., density), the extra-chromosomal genetic material with transmissibility may be horizontally transferred from donor to recipient (R), potentially accompanied by other mobilizable elements. Of note, this HGT may be amplified by the application of high-dose *B. thuringiensis*-based pesticides containing T^+^M^+^ characters, as illustrated in Fig. 1a.

Despite the success of *B. thuringiensis*-based formulations in pest management, their fate in the environment and effect on *B. cereus* group ecology are poorly understood. Furthermore, low efficiency and resistance development remain unsolved issues with such pesticides [17]. As the diversity and entomopathogenic origin of *B. thuringiensis* are mainly related to plasmids, understanding the effects of *B. thuringiensis*-based formulations on the plasmid pool and genetic structure of *B. cereus* group strains in the environment is essential for improving efficacy and resistance management.

In this study, we established confined niche plots to analyze the effects of single and mixed *B. thuringiensis* strain application on the plasmid pool and genetic structure of *B. cereus* group members. This study provides a basis for understanding the ecology of *B. thuringiensis*-based pesticides and their impact on the population structure of related group members, especially within confined habitats (e.g., greenhouse).

## Results and discussion

### Genetic background and transmissibility of the applied *B. thuringiensis*

The activity of the HD73 and 4AA1 strains against lepidopteran and coleopteran larvae is thought to be due to the presence of *cry1Ac* on pHT73 in the former and *cry3Aa* on pBM51 in the latter [15, 18]. We previously reported on the self-transfer and mobilization capabilities of pHT73 and pAW63 from the HD73 strain [15, 16]. The HD73 strain used in this study contains the conjugative plasmids pHT77 (*ori60-type*) and pHT73 (*ori44-type*) but lacks the conjugative plasmid pAW63 [19]. Thus, pHT73 was used as a positive control to determine the transmissibility (both self-transfer and mobilization) of plasmids in the 4AA1 strain. In addition, given the long history of mixed pesticides in pest resistance management [20], the effects of both single (HD73 or 4AA1) and mixed (HD73 + 4AA1) strain application on gene flow within the endogenous *B. cereus* group populations in sprayed habitats were studied. The 4AA1 strain contains six plasmids whose transmissibility potential has not yet been clarified [18]. Here, based on BLAST analysis, pBMB68, pBMB76, and pBMB232 carrying *ori43-*, *ori60-*, and *orf156*/*157-*type replicons, respectively, were predicted to be conjugative plasmid candidates as they all harbored genes encoding conjugal transfer proteins.

The polymerase chain reaction (PCR) results confirmed that the transconjugants grown on double-resistant plates produced by the tri-mating experiments using HD73 and 4AA1 as donors carried the mobilizing plasmid pBC16. This indirectly verified conjugative transfer and the presence of conjugative elements in both donors. Furthermore, 4AA1 displayed a slightly higher mobilizing capability than HD73, with frequencies of ca. 5.0 × 10^− 5^, 2.5 × 10^− 5^, and 1.5 × 10^− 5^ transconjugants/recipient in the Luria-Bertani (LB), soil, and leaf substrates, respectively. The mobility of mixed HD73 + 4AA1 (equal volumes) was between that of HD73 and 4AA1 alone, i.e., ca. 3.3 × 10^− 5^, 1.2 × 10^− 5^, and 8.0 × 10^− 6^ in LB, soil, and leaf media, respectively (Table 1). When using 4AA1 as the donor, only *ori60* was detected in the tested transconjugants carrying pBC16 (occurrence rate of 20 out of 45 tested transconjugants), with the *ori43-* and *orf156*/*157-*type replicons absent. When using the mixed donors, 14 of the 30 tested transconjugants carried *ori60* and none carried *ori44*-, *ori43-*, or *orf156*/*157-*type replicons. The absence of pHT73 in the tested transconjugants may be due to a frequency below the detection threshold (< 10^− 7^ transconjugants/recipient). In addition, our data suggested that pBMB76 may have competed with pHT73 during the mixed mating experiments. The dominance of pBMB76 during competition could be related to the “candidate” conjugative plasmids pBMB68 (*ori43-*type) and pBMB232 (*orf156*/*orf157*-type), which may have cooperated with pBMB76 or displayed a synergy for mobility. Indeed, recent research suggested a possible association between plasmids with *orf156*/*orf157* replicons and a specialized *B. thuringiensis* clade, which carries genes encoding toxins against Lepidoptera or Diptera, and that *orf156*/*orf157*-type plasmids may be important vectors or moderators of HGT [21]. In addition, some unknown genetic determinants located in pBMB76 itself, or some unknown chemical communication system in the 4AA1 host (like those in *Bacillus subtilis*, which are involved in the regulation of conjugation [22]), may be responsible for its higher efficiency compared to pHT73. However, this needs to be further clarified.

**Table 1:** Characteristics of the sprayed *B. thuringiensis* insecticides used in this study

*B. thuringiensis*	Identified self-conjugative plasmid type^a^	Mobility frequency^b^ (transconjugants/recipient)		
		LB medium	Soil	Leaf
HD73	*ori44*	1.3× 10^−5^ (6.5× 10^− 6^)	3.8 × 10^− 6^ (2.2× 10^− 6^)	3.7× 10^− 6^ (2.8× 10^− 6^)
4AA1	*ori60*	5.0 × 10^− 5^ (2.6× 10^− 5^)	2.5× 10^− 5^ (1.3× 10^− 5^)	1.5× 10^− 5^ (1.3× 10^− 5^)
HD73 + 4AA1	*ori60*	3.3× 10^−5^ (2.1× 10^− 5^)	1.2× 10^− 5^ (6.6× 10^− 6^)	8.0× 10^− 6^ (7.0× 10^− 5^)

^a^Only the occurrence of the replicons of *ori43*-, *ori44*-, *ori60*- and *orf156/157* -type plasmids which were predicted to carry conjugal gene(s) were surveyed in the transconjugants

^b^Average value of at least three independent mating experiments with SE in the bracket

### Effects of *B. thuringiensis* application on genetic transmissibility within *B. cereus* group populations in sprayed habitats

As the genetic backgrounds of the new isolates were unknown, the self-transfer capability of their endogenous plasmids could not be determined directly but was monitored based on their mobility to the T^−^M^+^-type strain (i.e., helper strain GBJ002 (pBC16) used in tri-mating experiments) [11]. In total, 684 isolates were identified from soil and leaf samples (average of 40/set) and used for the evaluation of genetic transmissibility. The percentages of isolates with mobility in the control sets were at a similar level (~ 12–21%). However, after the first application in Y1, the percentage of isolates with mobility in the HD73- and 4AA1-sprayed groups increased, with 4AA1 alone demonstrating the highest mobility (ca. 54%) in the leaf population. After application in Y2, the percentage of isolates with mobility in the HD73- and 4AA1-sprayed groups was 4–5 times higher than that in the controls (Fig. 1b). These results correspond to our hypothesis in Fig. 1a: i.e., if *B. thuringiensis* pesticides with transmissibility are sprayed in a confined habitat, some recipients will obtain conjugative and/or mobilizable plasmids and the resulting transconjugants may become new donors, resulting in a gradual increase in the proportion of T^+^M^+^-type strains in the habitat until saturation.

Unexpectedly, the ratio of isolates with mobility in the mixed group increased but was not higher than that of the single-sprayed groups, except for the Y1-soil-set (Fig. 1b). This was probably due to cooperation or competition among different types of conjugative plasmids, including known plasmids in HD73 and 4AA1 and unknown MGEs present in the environmental isolates. Thus, the use of HD73 and 4AA1 significantly promoted the HGT of genetic material within the applied habitats. However, the planting conditions in the study plots may not be representative of conditions within open natural environments as growth and interactions between donors and recipients can be affected by a multitude of natural factors, e.g., nutrient levels, rainfall, temperature, pH, UV light, and competition or cooperation with other symbiotic organisms.

### Distribution of plasmids related to transmissibility within *B. cereus* group populations in sprayed habitats

**Fig. 2:**
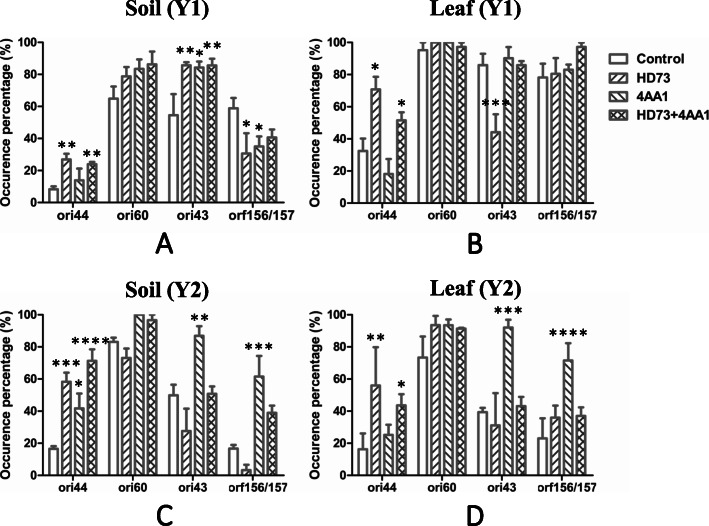
Distribution of tested plasmids with potential transmissibility in *B. cereus* group isolates in soil (**a**) and leaf (**b**) substrates in 2017 and soil (**c**) and leaf (**d**) substrates in 2018. In total, 684 isolates, (ca. 40/set) from soil and leaf substrates were tested. *P <* 0.1: *; *P <* 0.01: **; *P <* 0.001: ***; *P <* 0.0001: ****

Considering that the *ori44*- and *ori60*-type plasmids were the main contributors to the transmissibility and mobility of the HD73 and 4AA1 formulations, respectively, their occurrence rates were surveyed. The occurrence of *ori44* in the soil and leaf substrates of the Y1-HD73-sprayed group was three and two times higher than that of the control, respectively. Even greater increases were observed in Y2 (3.5-fold that of the control in both soil and leaf samples), corresponding to obviously higher mobility in Y2 than in Y1. Although the occurrence rates of *ori60* in the 4AA1- and mixed-sprayed groups also increased, they were not significant. This may be related to an already high endogenous presence of *ori60*-type plasmids in environmental isolates, even without application (Fig. 2). Moreover, although self-transfer of *ori43*- and *orf156*/*orf157*-type plasmids was not observed under lab conditions, the distributions of *ori43* and *orf156*/*orf157* in the isolates of the 4AA1-sprayed group increased markedly, except for the Y1-soil set, in which the endogenous presence of *orf156*/*orf157*-type plasmids was much higher in the control than in the treated groups. This suggests that the transfer of pBMB68 and pBMB232 or other endogenous *ori43*- and *orf156*/*orf157*-type plasmids may occur.

### Genetic structure of *B. cereus* group community

**Fig. 3:**
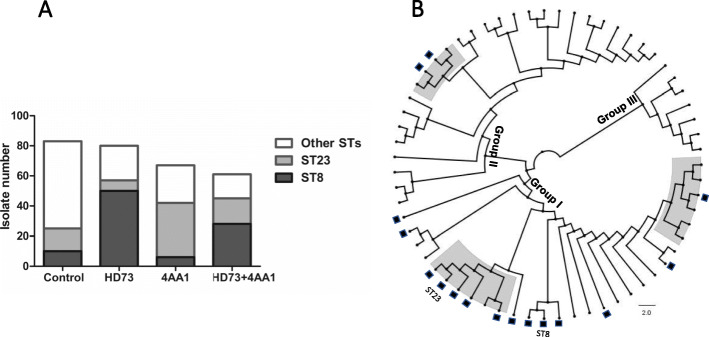
Genetic structure of *B. cereus* group isolates. **a** Distribution of STs in control and three treatment groups. Following MLST, 291 *B. cereus* group isolates were classified into 63 unique STs. **b** NJ phylogenetic tree based on concatenated sequences of seven loci. Clustering revealed three major groups (I, II, and III). STs containing isolates with mobility potential are marked using a black rectangle. Genealogical groups identified by goeBURST analysis at SLV level correspond to three subclades in dendrogram and are marked in shadow

In the sprayed plots, the ratio of isolates with mobility increased substantially. This was likely due to 1) the import and colonization of HD73 and 4AA1; and 2) the transfer of conjugative plasmids pHT73 and/or pBM76, or other plasmids with mobilizing capability, into isolates in the plot habitats, which then gained donor capability for further transfer and/or mobilization. To explore this, half of the isolates in each set were randomly selected for chromosomal background analysis. Altogether, 291 isolates were MLST sequenced and classified into 63 distinct sequence types (STs), including 37 in the control group, 13 in the HD73 group, 20 in the 4AA1 group, and 11 in the mixed group. Compared with that in the control group, the occurrence of other STs (i.e., non-ST8 or non-ST23) was dominant (ca. 70%). Specifically, in the HD73-sprayed group, 50 out of 80 sequenced isolates (62.5%) belonged to ST8, and in the 4AA1-sprayed group, 36 out of 67 sequenced isolates (53.7%) belonged to ST23 (Fig. 3a). These results indicate that the applied *B. thuringiensis* bacteria competed in the habitat with the resident bacteria and became dominant in the *B. cereus* group community.

In addition, although some ST8 and ST23 isolates shared identical STs, they carried different plasmid contents and *cry* genes (Table 2, Table S1). For instance, unlike HD73 itself or the ST8 isolates in the control group, which lacked *ori43*, the ST8 isolates in the treated groups occurred at a relatively high rate (16 out of 48 isolates in the HD73-sprayed group, and 19 out of 28 isolates in the mixed-sprayed group). Similarly, 25 out of 60 isolates belonging to ST23 in the treated groups carried *ori44*, which was absent in 4AA1 itself. Not all isolates carrying candidate conjugative plasmids were found to have mobility. The incidence rates of *ori44* and *ori60* in isolates belonging to ST8 were 72 and 95%, respectively, whereas the incidence rate was only 62% for those with mobility. In addition, ca. 30% of isolates belonging to ST8 carried *cry3A*, which was absent in HD73. Of note, although the occurrence of *cry1A* was generally high, even in the control group, this was not unexpected as *cry1* is predominantly located on MGE-associated cassettes in different plasmid types and carried by a variety of *B. thuringiensis* isolates [2]. Therefore, our results indicated the occurrence of plasmid flow within the community, and that enhancement of transmissibility was not only a consequence of clonal expansion but also likely due to the conversion of endogenous ST8 and ST23 isolates from non-mobile to mobile.

**Table 2:** The occurrence ratio of the isolates carrying the tested genes in the two dominant ST groups

Isolates		*cry1A*	*cry3A*	*ori43*	*ori44*	*ori60*	*orf156/157*	Mobility
ST8	Control-group	5/6	0/6	0/6	5/6	6/6	0/6	0/6
	HD73-group	36/48	17/48	16/48	36/48	46/48	13/48	30/40
	4AA1-group	1/6	1/6	5/6	1/6	6/6	2/6	1/3
	Mixed group	26/28	8/28	19/28	22/28	26/28	13/28	12/25
	Total	78%	30%	46%	72%	95%	32%	62%
ST23	Control-group	11/14	0/14	14/14	3/14	14/14	13/14	13/14
	HD73-group	2/7	0/7	4/7	3/7	5/7	3/7	5/7
	4AA1-group	25/36	8/36	33/36	13/36	35/36	29/36	33/35
	Mixed group	11/17	4/17	14/17	9/17	16/17	15/17	16/17
	Total	65%	16%	91%	37%	96%	81%	93%

The concatenated sequences of the seven loci from the 63 distinct STs were aligned and a neighbor-joining (NJ) phylogenetic tree was constructed. Clustering revealed three major groups. Remarkably, STs with mobility potential were mainly located in group I (ratio 14/16) (Fig. 3b), indicating that similar chromosomal backgrounds might be beneficial for genetic exchange. However, despite sharing identical or highly similar genetic backgrounds, not all those belonging to the same ST obtained conjugative plasmids. This is understandable as the acquisition of conjugal and mobilizing plasmids in natural habitats requires other physical conditions, such as close contact, and plasmid loss can also occur [23]. Alternatively, transmissibility failure could also be related to competition among endogenous plasmids, restriction modification, or other genetic defects [24].

GoeBURST analysis identified three genealogical groups at the single-locus variant (SLV) level, all of which carried at least one ST with mobility. Interestingly, ST23 was recognized as the ancestor of one genealogical group that consisted of six STs. Although ~ 25% of ST23 isolates were found in the control and HD73-sprayed habitats, almost all other isolates were found from the 4AA1- or mix-sprayed habitats, indicating that they may have diverged from ST23. However, phylogenic analysis did not explore more related evolutionary events due to the short timeline.

## Conclusions

Our study suggested that the application of *B. thuringiensis* in certain habitats could result in these bacteria becoming dominant in the *B. cereus* group community, thus promoting the transmission of MGEs related to insecticidal traits. In actual field application, *B. thuringiensis*-based formulations are usually sprayed at a lower dose but at a higher frequency than applied in the current study (ca. 1 × 10^9^ CFU/m^2^ and 2–3 times during each planting season) (see the Kernel Bio-Tech Co., Ltd. handbook). As *B. thuringiensis* can move from soil to aerial parts of plants and to the midgut of larvae [25], manipulating the dominant genetic population in soil by natural HGT may be a convenient way in which to reduce the usage and frequency of *B. thuringiensis* formulations. However, this may introduce issues related to resistance and biological diversity. How *B. thuringiensis* pesticides with transmissibility shape the genetic structure and diversity of *B. cereus* group populations in the natural environment is likely to be complicated. The role of HGT in adaptation to environmental and ecological interactions related to social cooperation and conflict, as well as its relationship with population genetic diversity, needs to be clarified.

## Methods

### Strains and media

The bacterial strains used in this study included *B. thuringiensis kurstaki* HD73 and *B. thuringiensis tenebrionis* 4AA1 (Table 1), with a chromosomal background of MLST ST8 and ST23, respectively. The two strains are frequently used to control lepidopteran and coleopteran larvae. Here, for tri-parental matings, *B. thuringiensis* subsp. *israelensis* GBJ001 and GBJ002 (pBC16) were used as the recipient and helper strains, respectively, to test the transmissibility and mobility of the *B. thuringiensis* pesticides and the environmental *B. cereus* group isolates. Both GBJ001 and GBJ002 are plasmid-cured derivatives of 4Q2 [26], with the former carrying streptomycin (Sm) resistance and the latter carrying nalidixic acid (Nal) resistance. In addition, pBC16, which is a mobilizable plasmid with tetracycline (Tet) resistance, was used to monitor the transfer of candidate conjugative plasmids in the environmental isolates without selective markers, as described previously [11]. Mannitol-egg-yolk-polymyxin (MYP) agar selective plates [27] and Luria-Bertani (LB) media were used for the isolation and growth of *B. cereus* group isolates [28]. Antibiotics were used at the following concentrations: Sm, 100 μg/ml; Nal, 15 μg/ml; and Tet, 10 μg/ml.

### Small-scale field experiment

Soil from a private farm located in a suburb of Wuhan (China), without a history of *B. thuringiensis* application, was collected, mixed, and evenly allocated into eight tub plots (100 × 75 × 15 cm). *Brassica campestris chinensis* individuals were planted in each tub and left to grow for 4–6 weeks without any pesticide application or disturbance in September 2017 (Y1) and September 2018 (Y2). Assigned formulations were then applied to the eight plots: two plots were sprayed with *B. thuringiensis kurstaki* HD73, two plots were sprayed with *B. thuringiensis tenebrionis* 4AA1, two plots were sprayed with an equal combination of the two formulations, and the last two plots were untreated to act as controls. The *B. thuringiensis* spray consisted of a combination of spore and vegetative cells at a ~ 4:1 ratio, with a total amount of 1.5–2.0 × 10^10^ CFUs/plot. Spraying was performed once for each planting season. The plots were then covered with nylon gauze to avoid cross contamination by flying pests but not to prevent exposure to natural sunlight and rain. The plots were placed in an open-air environment during the whole planting period. Soil and leaf samples were collected immediately before biopesticide application (D0), and at 6-d intervals after application from days 6 to 36 (D6–D36). For each treatment group, soil was collected from the top 1–2 cm of the surface in five spots in each plot (1 g/spot), then mixed and divided into three trials. Similarly, 10 separate leaves (ca. 5 × 5 cm), including emerging and mature leaves, were taken from each plot, mixed, and then divided into three trials. Sampling at different spots and intervals was used to disperse the samples as much as possible as clonal populations are prone to colonize at one site at the same time point. In total, 336 leaf and soil trials (= 16 sets × 7 time points × 3 trials) were established. Two to three randomly selected *B. cereus* group colonies per soil or leaf sample were streaked and stored.

### Tri-parental mating experiments

Tri-parental mating was used to investigate transmissibility and mobility, as described previously [11]. The conjugation experiments were conducted as per earlier research [5]. Mating in liquid LB or soil was performed with a similar method. Briefly, equal amounts of donor, helper, and recipient strains were mixed and added into fresh LB broth or onto the surface of sterilized soil (2 g) in 15-mL glass tubes. The mixtures were incubated at room temperature without shaking for 4 h. The former was then plated directly onto selective media at an appropriate dilution, and the latter was dispersed using 5 mL of ddH_2_O before plating. Mating on the phyllosphere was performed using the drop-on-drop method. In brief, overnight pre-cultures of donor, helper, and recipient cells (10 μL each) were mixed and placed on the same spot of a sterilized leaf piece (2 × 2 cm) and incubated at room temperature for 4 h. After mating, the resulting colony was washed from the leaf before plating. Transconjugants containing mobilizable plasmids were first screened on double-resistant plates (Sm^R^Tet^R^) and then verified by polymerase chain reaction (PCR). Donor, helper, and recipient strain controls were plated on selective media to exclude the possibility of spontaneous resistant mutants. Each mating experiment was repeated at least three times independently, and transfer frequencies were calculated as the ratio of transconjugants to recipient cells (T/R).

For the *B. thuringiensis* pesticides, to simulate the conjugation phenomenon in natural habitats, mating experiments were performed in LB medium as well as in soil and on the phyllosphere. For the new isolates, mating was only performed in LB medium. In total, 684 isolates (average 40/set) from soil and leaves were tested for mobility.

### Primers, PCR screening, and MLST

The primers used in this study are listed in Table S2. Of these, Bcg-F/R, which is specific to the *B. cereus* group [29], was used to check the isolates screened by the MYP selective plates, and primer pairs Ori43_F/R, Ori44_F/R, and Ori60_F/R were used to determine the occurrence of potential plasmids with transmissibility. In addition, primer pair Bti1_for/Bti1_rev, which is specific for *B. thuringiensis israelensis* [29], was used to verify the chromosomal background specific to the recipient, and primer pair pBC16-F/pBC16-R was used to confirm the presence of pBC16. The MLST scheme and primers were used as described in the PubMLST database (http://pubmlst.org/bcereus/), which assigns sequence type (ST) based on seven housekeeping genes (i.e., *glp*, *gmk*, *ilv*, *pta*, *pur*, *pyc*, and *tpi*) [30]. Amplifications were carried out on a Heal Force Thermal Cycler (ETC811, Eastwin Inc., China) using GoTaq DNA Polymerase according to the protocols provided by the reagent company (*Promega* (Beijing) Biotech Co., Ltd., China). In total, 291 isolates randomly selected from the different treatment sets were sequenced, producing 63 unique STs.

### Phylogenic analysis

Using MEGA 6.0 [31], the 63 concatenated DNA sequences of seven loci were aligned and a neighbor-joining (NJ) phylogenetic tree was constructed based on the maximum composite likelihood model with 1000 bootstraps. Furthermore, goeBURST v1.2.1 [32] was used to identify the genealogical relationships of bacilli representing different STs based on single-locus variants (SLV).

### Statistical analysis

Two-way analysis of variance (ANOVA) was used to analyze the proportion of mobility and replicon genes, with the sprays set as fixed factors and enclosure set as a random factor. The model assumptions (normality, homoscedasticity, and error distribution) were graphically analyzed using GraphPad Prism 6.0 (www.graphpad.com). The indicated significance (*P <* 0.1) in the analysis was tested again via sequential deletion of sets from the full model.

## Supplementary Information

**Additional file 1: Table S1**. Detailed characteristics of 291 *B. cereus* group isolates sequenced for MLST analysis.

**Additional file 2: Table S2**. Primers used in this study.

**Additional file 3: Figure S1**. GoeBURST analysis at SLV level. Scattered dots without assigned number are new STs.

## Data Availability

All data generated or analyzed in this study are included in the published article [and its supplementary information files].

## Abbreviations

ANI: Average nucleotide identity
ICPs: Insecticidal crystalline proteins
MGEs: Mobile genetic elements
MLST: Multi-locus sequence typing
PFGE: Pulsed field gel electrophoresis
HGT: Horizontal gene transfer
ST: Sequence types
