# Supplementary material for: Application of Bacillus thuringiensis strains with conjugal and mobilizing capability drives gene transmissibility within Bacillus cereus group populations in confined habitats
Source: BMC Microbiol. 2020 Nov 26;20:363. doi: 10.1186/s12866-020-02047-4 (PMC7690115; doi:10.1186/s12866-020-02047-4)
Supplement: Supplementary file 1 — Additional file 1: Table S1. Detailed characteristics of 291 B. cereus group isolates sequenced for MLST analysis. [file 12866_2020_2047_MOESM1_ESM.docx]

Table S1. Primers used in this study

| Primer | Target/description | Sequence (5’-3’) |
| --- | --- | --- |
| Bti1_for | Specific for *B. thuringiensis israelensis* | CAAACATTTCATTCCAATAACA |
| Bti1_rev |  | ATACTGTGTGGGATGCTTATTA |
| Bcg-F | Specific for *B. cereus* group strains | AACAGGCTCCATACAATGGTAT |
| Bcg-R |  | TGGTAGCGTTTCTTCGTCTTAT |
| Ori43_F | *ori43*-type plasmid | tgacacactggacaccacaa |
| Ori43_R |  | CCCCAAGATCYGCTTCTAAT |
| Ori44_F | *ori44*-type plasmid | GTGATAGCCCAAAAAGCGGG |
| Ori44_R |  | TCAAAACGATCGGCAGCTTG |
| Ori60_F | *ori60*-type plasmid | ggccaaacgcctacctttat |
| Ori60_R |  | tgtccagctcttgcatgttc |
| ORF156_F | *orf156/157*-type plasmid | cgccaaatccttggttttta |
| ORF156_R |  | GGATTCTTCTCAGCRGAACA |
| cry1A_F | *cry1A* | CCGGTGCTGGATTTGTGTTA |
| cry1A_R |  | AATCCCGTATTGTACCAGCG |
| cry3A_F | *cry3A* | TGCAGAGTTACAGGGCCTTC |
| cry3A_F |  | GGAACCGCGTGTGAAATTGA |

(Y=C or T; R=A or G).
