# Supplementary figures and images for: Application of Bacillus thuringiensis strains with conjugal and mobilizing capability drives gene transmissibility within Bacillus cereus group populations in confined habitats
Source: BMC Microbiol. 2020 Nov 26;20:363. doi: 10.1186/s12866-020-02047-4 (PMC7690115; doi:10.1186/s12866-020-02047-4)

## Slide 1
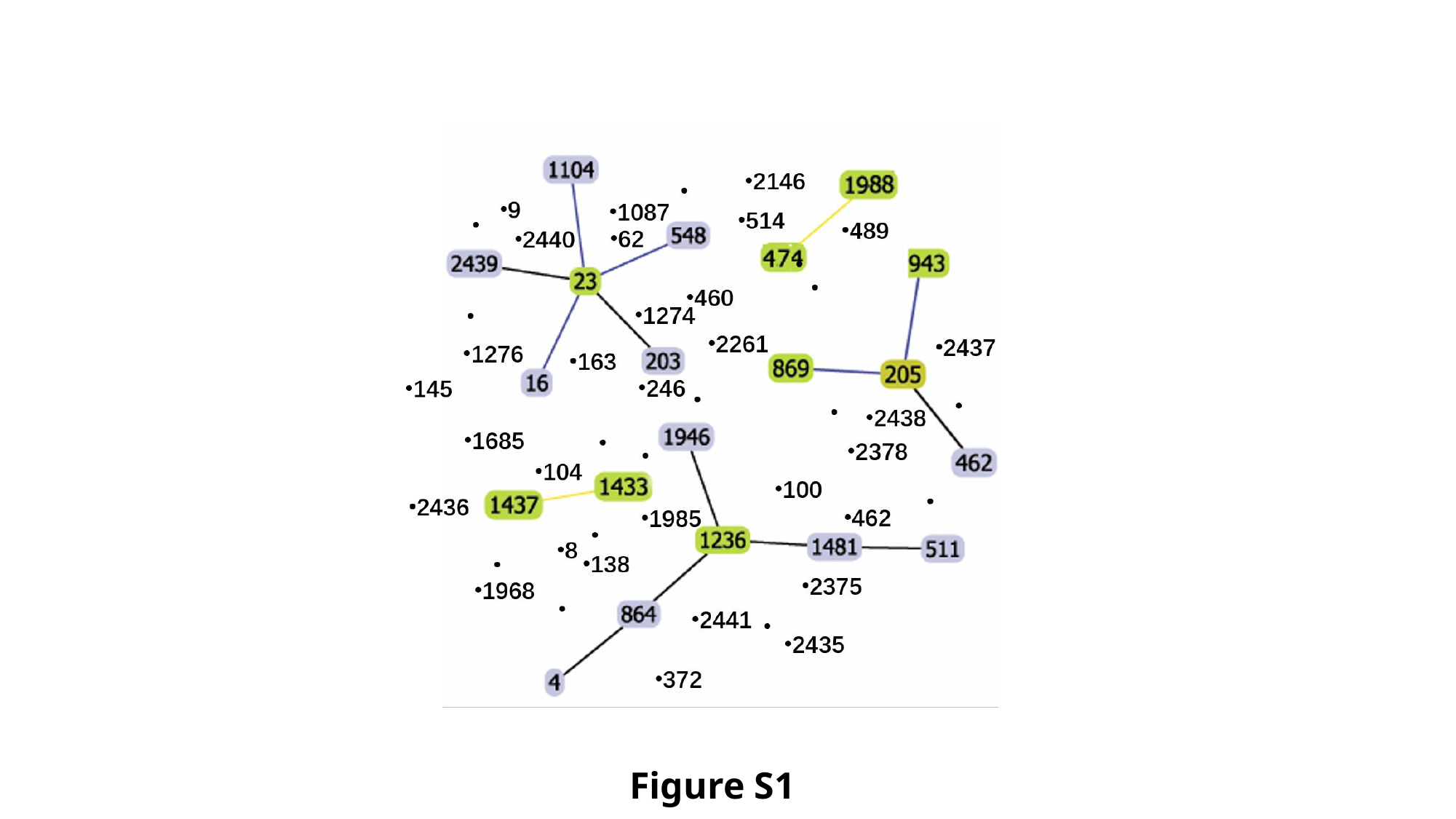

Figure S1

Supplement: Supplementary file 3 — Additional file 3: Figure S1. GoeBURST analysis at SLV level. Scattered dots without assigned number are new STs. [file 12866_2020_2047_MOESM3_ESM.pptx]
